# Vertical traction for lumbar radiculopathy: a systematic review

**DOI:** 10.1186/s40945-021-00102-5

**Published:** 2021-03-15

**Authors:** Carla Vanti, Luca Turone, Alice Panizzolo, Andrew A. Guccione, Lucia Bertozzi, Paolo Pillastrini

**Affiliations:** 1 Department of Biomedical and Neuromotor Sciences (DIBINEM), Alma Mater Studiorum, University of Bologna, 40138 Bologna, Italy; 2 Department of Rehabilitation Science, College of Health and Human Services, George Mason University, Fairfax, VA 22030 USA; 3 School of Physical Therapy, Alma Mater Studiorum University of Bologna, Bologna, Italy

**Keywords:** Traction, Sciatica, Radiating pain, Low Back pain, Pain management, Intervertebral disc disease, Randomized controlled trials, Disability evaluation

## Abstract

**Background:**

Only low-quality evidence is currently available to support the effectiveness of different traction modalities in the treatment of lumbar radiculopathy (LR). Yet, traction is still very commonly used in clinical practice. Some authors have suggested that the subgroup of patients presenting signs and symptoms of nerve root compression and unresponsive to movements centralizing symptoms may benefit from lumbar traction. The aim of this study is to conduct a systematic review of randomized controlled trials (RCTs) on the effects of vertical traction (VT) on pain and activity limitation in patients affected by LR.

**Methods:**

We searched the Cochrane Controlled Trials Register, PubMed, CINAHL, Scopus, ISI Web of Science and PEDro from their inception to March 31, 2019 to retrieve RCTs on adults with LR using VT to reduce pain and activity limitation. We considered only trials reporting complete data on outcomes. Two reviewers selected the studies, extracted the results, and performed the quality assessment using the Risk of Bias and GRADE tools.

**Results:**

Three studies met the inclusion criteria. Meta-analysis was not possible due to the heterogeneity of the included studies. We found very low quality evidence for a large effect of VT added to bed rest when compared to bed rest alone (g = − 1.01; 95% CI = -2.00 to − 0.02). Similarly, VT added to medication may have a large effect on pain relief when compared to medication alone (g = − 1.13; 95% CI = -1.72 to − 0.54, low quality evidence). Effects of VT added to physical therapy on pain relief were very small when compared to physical therapy without VT (g = − 0.14; 95% CI = -1.03 to 0.76, low quality evidence). All reported effects concerned short-term effect up to 3 months post-intervention.

**Conclusions:**

With respect to short-term effects, VT may have a positive effect on pain relief if added to medication or bed rest. Long-term effects of VT are currently unknown. Future higher quality research is very likely to have an important impact on our confidence in the estimate of effect and may change these conclusions.

**Supplementary Information:**

The online version contains supplementary material available at 10.1186/s40945-021-00102-5.

## Background

Low back pain (LBP) is a common musculoskeletal disorder [1]. Most LBP are non-specific, and only 3–5% of the general population is affected by lumbar radiculopathy (LR) [2], that is a pain syndrome caused by compression and/or irritation of lumbar nerve roots [3]. LR is a common reason for physician consultations and imaging referrals [4]; typical symptoms are radiating pain, often with numbness, paresthesia, and/or muscle weakness [4].

The initial management for LR is conservative treatment, as recommended by the North American Spine Society [5]. Among different interventions [6], lumbar traction has been used for decades in the treatment of acute or chronic LBP [7], with or without sciatica [8, 9]. Delitto [10] and Fritz [11] have suggested that the subgroup of patients presenting signs and symptoms of nerve root compression and who are unresponsive to movements that centralize symptoms may benefit from lumbar traction.

On the contrary, previous reviews have not confirmed the effectiveness of traction for LBP, with or without LR [6, 12, 13]. Judging the state of the literature as a whole, only low-quality evidence is currently available supporting the effectiveness of different traction modalities in the treatment of LR [12]. Despite this gap in knowledge, it is very commonly used in clinical practice [7, 14].

Traction can be manual or mechanical, and traction forces may be applied continuously (maintained for 20 min or more) or intermittently (alternating traction and relaxation with cycles of a few minutes each) [12]. Traction rhythm, force, and patient position can also vary.

Vertical traction (VT) exerts a distractive force by suspending the patient while held in a vertical or seated position using a belt around the chest or placing the patient in an upside down position from the ankles (the so-called inverted lumbar traction) [15]. Patients may also be asked to perform traction independently by using a pull-up bar to suspend the trunk in vertical position. Finally, VT may be done in water, using the same modalities previously described with the addiction of an external weight placed on the patient’s ankles [16]. As a result, traction force can vary from upper half patients’ body weight plus gravity to a patient’s full body weight plus gravity and/or external weight [17].

Traction forces in VT are likely to be more consistent and tailored to each patient than manual traction. In fact, they are linearly proportional to the weight of the patient’s lower body. Using the second law of Newton, the force exerted on the lower disk spaces while in suspension can be calculated with the formula F = m x g, where F is force with the unit of Newton, m is the weight of the lower body in kilograms, and g is a constant for the gravity of earth, which is equal to 9.8 [18]. Gravitational traction produces a greater widening of the individual disc space than the static supine lumbar traction and may result in decreased intradiscal pressure and pain [19]. However, most traction devices are available only in physical therapy clinics, which places a burden on patients to receive treatment.

Globally, researchers emphasize the need to identify targeted delivery methods of traction that match appropriate parameters and patient populations [12]. For all these reasons, we conducted a systematic review to investigate the effectiveness of each different type of VT compared with or added to other conservative treatments on pain and activity limitations, in patients with LR.

## Main text

### Methods

This systematic review protocol was registered in the PROSPERO database (code CRD42019136591) and followed PRISMA recommendations (see Additional file 1).

#### Data sources and searches

The authors undertook a multiple database electronic search of articles in the following databases: Cochrane Controlled Trial Register, PubMed, CINAHL, Scopus, ISI Web of Science, and PEDro. The following search terms in various combinations were utilized: “sciatica”/"radiculopathy”/"radicular syndrome”/“nerve root pain”/"leg pain/"low back pain”; “traction”/"physical therapy modalities” and adapted for the search in all databases (see Additional file 2).

Databases were searched from their inception until March 31, 2019. Additional records were explored by manually searching reference lists of selected articles, systematic reviews and Guidelines on LBP and LR, and personal records of the authors. If necessary, authors were contacted for missing information. Two independent blinded reviewers (AP, LT) conducted study selection. They first imported all results on EndNote X9 to search for and delete duplicates [20], and then they screened titles, abstracts and full texts using Rayyan QRCI [21]. Systematically the two authors compared their results; when disagreement occurred, a third expert author (CV) was consulted.

#### Studies selection

We included in this systematic review randomized controlled trials (RCT) on humans, in all languages and published in every date. We included only studies on adults (≥18 years) with LR confirmed by the presence of at least two of the following criteria:

1. radicular symptoms: LBP with pain and/or numbness radiating below the knee.
2. ≥1 radicular signs:
  - sensory loss/paresthesia in any of the L4-S1 dermatomes;
  - diminished Patellar/Achilles reflex;
  - muscle strength deficit in any of the L4-S1 myotomes.
3. positive imaging (MRI/CT) [5].

Trials including patients without signs and symptoms of LR were excluded; trials involving patients with other specific diagnoses/current pregnancy/early postpartum period were also excluded. We included RCTs in which every type of VT was applied alone or in combination with other conservative or pharmacological treatments. Only trials with complete data regarding traction (patient position, traction type, rhythm, force, duration and frequency; and number of sessions) were considered for inclusion. We considered every type of non-traction therapy, including other conservative treatment, placebo, sham treatment, minimal care, or no intervention, a control/comparison group as long as traction was the main contrast between intervention and control group. We excluded studies comparing different traction types or traction parameters.

We considered as primary outcome the intensity of pain perceived in lumbar and/or sciatic areas, measured with a Numerical Rating Scale (NRS) or a Visual Analogue Scale (VAS), In case of separate data concerning lumbar and sciatic pain, we selected those about leg pain. Secondary outcomes were: physical functioning, measured with the Oswestry Disability Index (ODI) or the Roland & Morris Disability Questionnaire (RMDQ); lumbar/leg mobility; psychological parameters (e.g. fear-avoidance beliefs, depression, anxiety); quality of life; changes in neurological function (e.g. Straight Leg Raising, Herniation Index, etc.); and treatment adherence. In cases in which data were lacking, we contacted the authors to obtain them. When they were impossible to obtain, we estimated unreported standard deviations borrowing them from one or more other studies [22].

Adverse effects, when reported, were collected.

Outcome measures were collected at short-time (up to 3 months), mid-time (from three to 6 months), and long-time (more than 6 months) follow-ups.

#### Data extraction and quality assessment

Two reviewers (AP, LT) independently conducted data extraction and collection of the following data:

- population: total number of participants; number of participants of treatment/control groups; proportion of male/females; mean age; previous episodes of LBP; mean pain intensity; mean physical functioning; number of patients taking drugs;
- interventions: setting and geographical area where the intervention was conducted; type of intervention, with frequency, intensity, number/duration of sessions;
- comparisons: type of control, including frequency, intensity, number/duration of sessions;
- outcomes: measurement tools used to record each outcome; means and standard deviations of each outcome at the baseline and each follow-up for all groups; measurement scales/questionnaires and their direction for each outcome.

For any disagreement, another expert author (CV) was consulted.

The same two authors conducted the risk of bias assessment using the Cochrane Collaboration’s Risk of Bias (RoB) Tool [23, 24]. This tool comprises 13 items, each item was scored as “YES” if it fulfilled the criterion, “NO” if there was a clear RoB, and “UNSURE” if there was insufficient information. To summarize the overall RoB for a study, according to Gianola and colleagues, items related to allocation concealment, blinding of outcome assessment, and incomplete outcome data were considered [25]. Studies were classified as at “low risk of bias” when all three criteria were met, at “high risk of bias” when at least one criterion was unmet, and at “moderate risk of bias” in the remaining cases.

We evaluated the overall quality of evidence using the Grading of Recommendation, Assessment, Development and Evaluation (GRADE) framework [26]. GRADE is a systematic and explicit approach to make judgments about quality of evidence and strength of recommendations. Using GRADE, we rated the evidence not by individual study, but across studies for specific clinical outcomes. We considered the five GRADE domains:

1. study limitations for RoB assessment was defined “serious” if studies were classified as “high risk of bias” or “not serious” if studies were classified as “moderate/low risk of bias” using the Cochrane Collaboration’s Risk of Bias (RoB) Tool [23, 24];
2. inconsistency, in case of conflicting results;
3. indirectness, to describe comparisons of the characteristics of population, setting and outcomes to those of our clinical practice;
4. imprecision, identifying studies that include relatively few patients and few events and thus have a wide confidence interval around the estimate of the effect [27];
5. publication bias, describing the possibility that a systematic under-estimation or over-estimation of the underlying beneficial or harmful effect is due to selective publication [28].

We planned to perform the assessment of publication bias using the Egger t test only if ten or more studies have been included in our systematic review. The test proposed by Egger in 1997 may be used to test for funnel plot asymmetry. General considerations suggest that the power will be greater in the continuous outcomes than for dichotomous outcomes, but that use of the method with substantially fewer than 10 studies is unwise [22].

We decided to evaluate the quality of the evidence using GRADE approach [29], even in the case when only a single RCT addressed a comparison, by careful scrutiny of all relevant issues (risk of bias, imprecision, indirectness, and publication bias) as suggested by GRADE Guideline [30].

#### Data synthesis and analysis

To calculate the effects of interventions, separate analyses were made according to traction type, patient position (vertical, sitting) and force delivered. We did not take into consideration the stage of LR, so that studies on subjects in acute (less than four weeks duration), subacute (from four to 12 weeks duration), or chronic (more than 12 weeks duration) stages of LR were analyzed together. Likewise, no differences in the statistical analysis were made regarding period of application of the traction therapy (continuous or intermittent).

We provided a descriptive synthesis of the findings and estimated the effects of interventions from the included studies. When possible, we calculated Hedge’s g using a random effects model to give a more conservative estimate of effect. We chose a priori to use the random effects method because it is a more conservative approach that also allows generalization of findings beyond the studies included in the synthesis. We used the Q and I-square statistics to assess heterogeneity across studies [31].

For statistical analysis we used the software ProMeta v.2.0 (Internovi by Scarpellini Daniele s.a.s., Cesena [FC], Italy; now owned by Idostatistics) [32]. We calculated standardized mean differences (SMDs) with 95% confidence intervals (95% CIs) for continuous data. To interpret effect size calculated with SMD, we used Cohen’s interpretations of d thresholds as a guide to identify very small (*< 0.20),* small (*≥0.20 < 0.50*), medium (*≥0.50 < 0.80*), or large (*≥0.80*) effects [32]. We calculated the effect size based on the reported data (means/standard deviations/sample sizes of intervention and control groups). We excluded the studies for which these or other essential data were not reported or obtainable by contacting authors.

## Results

### Characteristics of the selected studies

**Fig. 1:**
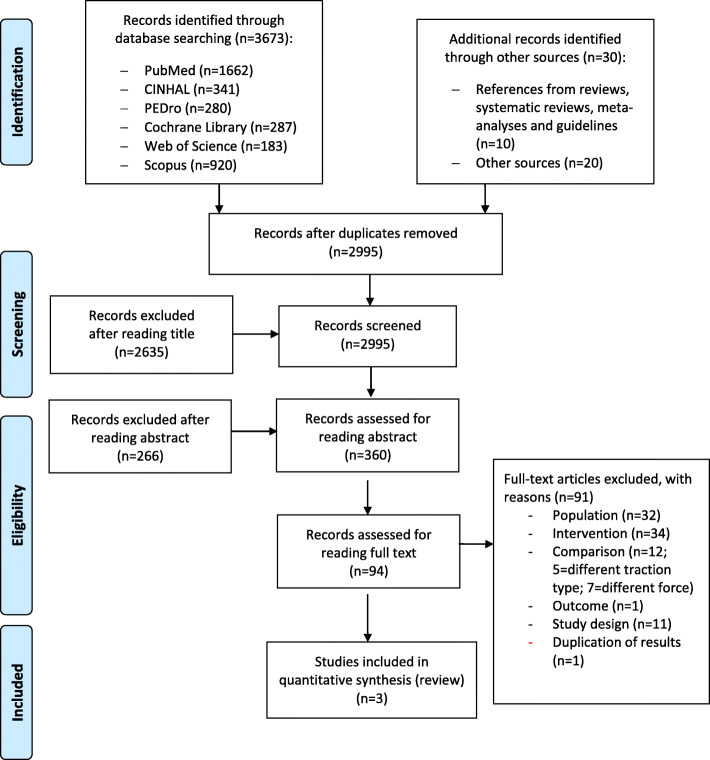
Flow diagram of included studies

We identified 3673 records through database searching and 30 additional studies through other sources, for a total of 3703 records. After we removed duplicates, we assessed 2995 records by title and abstract, of which 94 studies were eligible to be assessed by full text reading to verify the eligibility for inclusion in this systematic review. Of these 94, we excluded another 91 studies for different reasons (see Additional file 3), resulting in three studies available for quantitative synthesis (Fig. 1).

The three included studies were published from 1998 to 2015 and conducted in Iran [33], Nederland [34], and United Kingdom [35]. The total number of patients enrolled in the studies was 90; the total number of patients who completed the assessments was 85 (range 16–50), with a mean of 28.3 participants.

The different types of traction used were VT [33, 34] and inversion traction [35], which was considered a different type of VT for statistical analysis purposes. Two studies used intermittent traction [33, 35] and one study [34] used continuous traction. The duration of the treatments ranged from 1 week to 2 months and the duration of each treatment ranged from 10 to 45 min. Traction force ranged from upper half of patient’s bodyweight to patient’s bodyweight. Interventions with which traction were compared were physical therapy (PT) [35], medications [33], and bed rest [34].

Given the aims of this study, pain was considered as primary outcome measure. Concerning secondary outcome measures, we considered only activity limitation for quantitative analyses because it was reported in most of the selected studies [34, 35]. We included other outcomes collected in single studies (i.e. lumbar range of motion, global perceived recovery, Herniation Index, etc.) only in qualitative analyses.

All studies evaluated pain and activity limitation only at short-term follow-up. All the details regarding characteristics of the studies are shown in Table 1.

**Table 1:** Characteristics of Selected Studies

	Inclusion criteria	Exclusion criteria	Diagnostic criteria	Groups	Treatment	Outcome measures	Results as reported by the authors
**Khani et al** [33] **(2015) Iran**	- LBP with L3-S1 radiculopathy; - duration of symptoms < 6 months; - positive MRI findings; - no history of previous physical therapy; - willing to take part in the study by signing a written informed consent.	- red flags indicative of non-mechanical LBP; - indication of surgery; - spinal stenosis; - pregnancy or post-partum period.	Symptoms + physical exam	50 patients, randomized in 2 groups: ● Traction group (*n* = 25); ● Control group (*n* = 25).	2 months duration. 1. Autotraction in *vertical position* by suspension from a pull-up bar + routine medication ➔ **Traction rhythm**: continuous traction; ➔ **Traction frequency**: at least 20 times a day, each time for 30 s (or more than it so that the total duration of suspension reached 10 mins in a day); ➔ **Traction force**: patients’ body weight + gravity; ➔ **Combination with other interventions**: YES: Drugs (NSAID, Corticosteroids, Muscle relaxants). 2. Routine medication (NSAIDs, corticosteroids and muscle relaxants).	At baseline and after treatment: - pain (VAS); - Herniated Index (MRI).	VAS and Herniated Index reduction post-treatment in the traction group were significant. The clinical effect of pull-up bar traction is substantial.
**Moret et al** [34] **(1998) The Nederlands**	- age between 18 and 60 years; - LBP with radiculopathy; - at least two positive signs of radiculopathy: loss of sensitivity, paralysis in the musculature, provocation of symptoms with coughing or sneezing, positive SLR; - prescription of bed rest for at least 1 and a maximum of 2 weeks.	- signs of non-mechanical LBP with radiculopathy; - anatomical abnormalities (e.g. trunk-obesity, etc.); - any disease which may be a contraindication for traction therapy.	Symptoms + physical exam	16 patients randomized in 2 groups: ● Vertical traction + bed rest group (*n* = 8); ● Bed rest (toilet visits were allowed but must be registered in a diary) only group (*n* = 8).	At least 1 week and maximum 2 weeks. 1. Vertical traction in *sitting position* with a belt around the chest + bed rest ➔ **Traction rhythm**: continuous traction; ➔ **Traction frequency**: 4 times for 45 mins or 6 times for 30 mins per day; ➔ **Traction force**: patients’ body weight + gravity. ➔ **Combination with other interventions**: YES: rest on bed. 2. Bed rest.	At baseline and after 3 weeks: - activity limitation (RMDQ); - pain in the leg and in the back (10 point rating scale). In addition, at baseline and after 2 weeks: - Global Perceived Recovery; - Schöber score; - SLR.	RMDQ mean improvement and mean leg pain reduction were higher in the traction group. More patients in the study group strongly improved/completely recovered their back pain. No differences were found regarding global perceived recovery. SLR improved more in the traction group No differences were found in Schöber score improvement.
**Prasad et al** [35] **(2012) United Kingdom**	- age between 18 and 45 years; - duration of symptoms < 6 months; - signs and symptoms of single level unilateral radiculopathy with decision to operate.	- red flags indicative of non-mechanical LBP; - pregnancy or postpartum period; - increasing neurological deficits; - weight more than 20% of ideal norms for height and age or > 140 kg; - positive MRI findings.	Symptoms + imaging	24 patients randomized in 2 groups: ● Inversion therapy group + PT (*n* = 13); ● PT only (*n* = 11). Only 22 were eligible for assessment.	4 weeks. 1. Inversion traction in *vertical position* + PT (education and advice, exercises for movement control and reduction of derangement, and manual therapy) ➔ **Traction rhythm**: intermittent traction; ➔ **Traction frequency**: 3 times a week, 6 times 2-min inversion within tolerance; ➔ **Traction force**: upper half patients’ body weight + gravity; ➔ **Combination with other interventions**: YES: Physical Therapy (education, motor control exercises for derangement reduction, manual therapy). 2. PT.	At baseline and after 6 weeks: - activity limitation (RMDQ); - activity limitation (ODI); - pain (VAS).	Surgical intervention was avoided in 76.9% of patients in the inversion group, while it was avoided in only 22.2% in the control group. Patients in the inversion group tended to have less activity limitation at follow-up.

*LBP* Low Back Pain, *MRI* Magnetic Resonance Imaging, *NSAIDs* Non-steroidal Anti-inflammatory Drugs, *ODI* Oswestry Disability Index, *RMDQ* Roland & Morris Disability Questionnaire, *ROM* Range of Motion, *PT* Physical Therapy, *SLR* Straight Leg Raising, *VAS* Visual Analogue Scale

### Quality assessment

**Fig. 2:**
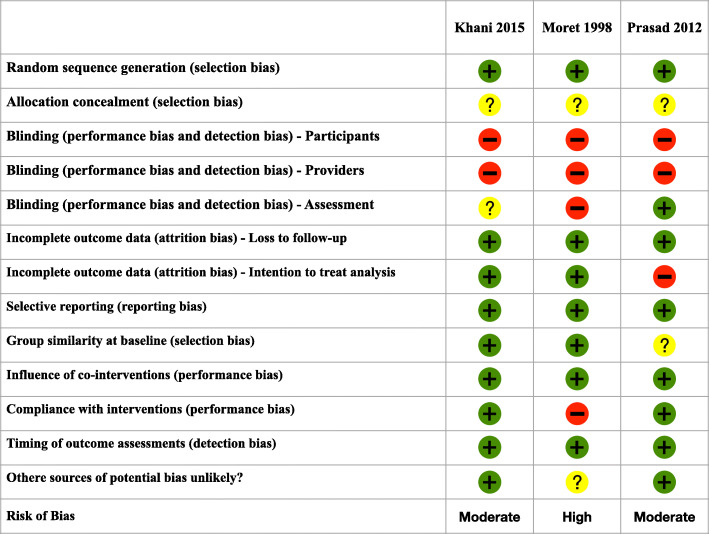
Risk of Bias of included studies according to the summarizing proposed by Gianola S. et al. [25]

Risk of bias assessment according to the Cochrane Collaboration’s Risk of Bias Tool [22] showed that two studies had moderate RoB [33, 35], and one had high RoB [34]. A complete description on RoB assessment is shown in Fig. 2.

### Effects of interventions

Table 2 shows the study findings for pain and activity limitation with respect to the effect size for intervention outcomes, with 95% CI values.

**Table 2:** Results of comparisons

OUTCOME	NO. OF STUDIES	NO. OF PARTICIPANTS	EFFECT SIZE. REPORTED AS HEDGES ***G*** (95% CI)	***p***
**VERTICAL TRACTION COMBINED WITH PHYSICAL THERAPY VERSUS PHYSICAL THERAPY ALONE.**				
Pain	1(Prasad 2012 [35])	19^a^	− 0.14 (− 1.03 to 0.76)	0.76
Activity limitation	1(Prasad 2012 [35])	19^a^	−0.31 (− 1.21 to 0.58)	0.49
**VERTICAL TRACTION COMBINED WITH BED REST VERSUS BED REST ALONE.**				
Pain	1(Moret 1998 [34])	16	−1.01 (−2.00 to −0.02)	0.04
Activity limitation	1(Moret 1998 [34])	16	−0.56 (−1.50 to 0.39)	0.24
**VERTICAL TRACTION COMBINED WITH ROUTINE MEDICATION VERSUS ROUTINE MEDICATION ALONE**.				
Pain	1(Khani 2015 [33])	50	−1.13 (− 1.72 to −0.54)	0.00

^a^ Prasad and colleagues recruited 26 patients, 24 of them were randomized, and 22 were eligible for assessment, but data on only 19 patients were published for the outcomes of pain and activity limitation. For this reason, only 19 participants were considered included

### First comparison: intermittent inversion traction combined with physical therapy versus physical therapy alone

#### Pain

Only one study [35] assessed this outcome and only at short-term follow-up. The effect size of VT was very small and non-significant (g = − 0.14) with a 95% CI from − 1.03 to 0.76. Following GRADE criteria this outcome provides low-quality evidence.

#### Activity limitation

Only one study [35] assessed this outcome and only at short-term follow-up. The effect size of VT was small and non-significant (g = − 0.31) with a 95% CI from − 1.21 to 0.58. Following GRADE criteria this outcome provides low-quality evidence.

### Second comparison: continuous VT combined with bed rest versus bed rest alone

#### Pain

Only one study [34] assessed this outcome and only at short-term follow-up. The effect size of VT was large and significant (g = − 1.01) with a 95% CI from − 2.00 to − 0.02. Following GRADE criteria this outcome provides very low-quality evidence.

#### Activity limitation

Only one study [34] assessed this outcome and only at short-term follow-up. The effect size of VT was medium and non-significant (g = − 0.56) with a 95% CI from − 1.50 to 0.39. Following GRADE criteria, this outcome provides very low-quality evidence.

### Third comparison: intermittent VT combined with routine medication versus routine medication alone

#### Pain

Only one study [33] assessed this outcome and only at short-term follow-up. The effect size of VT was large and significant (g = − 1.13) with a 95% CI from − 1.72 to − 0.54. Following GRADE criteria this outcome provides low-quality evidence.

The certainty of the evidence (GRADE) for each comparison is shown in Table 3.

**Table 3:** GRADE evaluation: quality of evidence and strength of recommendations

Quality						Summary of findings		
Outcome (No. of studies)	RoB^**a**^	Inconsistency	Indirectness	Imprecision^b^	Publication bias^c^	No. of participants	Effect size (SMD) with CI^d^	GRADE
**Vertical Traction + Physical Therapy VS Physical Therapy**								
**Pain ST (1)** (Prasad 2012 [35])	Not Serious	Not Serious	Not Serious	Serious	Likely	19^e^	−0.14 (−1.03 to 0.76)	LOW
**Activity limitation ST (1)** (Prasad 2012 [35])	Not Serious	Not Serious	Not Serious	Serious	Likely	19^e^	−0.31 (−1.21 to 0.58)	LOW
**Vertical Traction + Bed Rest VS Bed Rest**								
**Pain ST (1)** (Moret 1998 [34])	Serious	Not Serious	Not Serious	Serious	Likely	16	−1.01 (−2.00 to −0.02)	VERY LOW
**Activity limitation ST (1)** (Moret 1998 [34])	Serious	Not Serious	Not Serious	Serious	Likely	16	−0.56 (−1.50 to 0.39)	VERY LOW
**Vertical Traction + Medications VS Medications**								
**Pain ST (1)** (Khani 2015 [33])	Not Serious	Not Serious	Not Serious	Serious	Likely	50	−1.13 (−1.72 to −0.54)	LOW

^a^ RoB was considered “serious” in case of high risk of bias

^b^Imprecision was considered “serious” in case of one level (− 1) downgrading

^c^ Publication bias was not excluded, therefore it was considered sufficient for downgrading the quality of evidence

^d^ Effect size: Treatment effects favoring conservative intervention assigned negative Hedges standardized mean difference (SMD) values. ST = Short term

^e^ Prasad and colleagues recruited 26 patients, 24 of them were randomized, and 22 were eligible for assessment, but data on only 19 patients were published for the outcomes pain and activity limitation. For this reason, only 19 participants were considered included

### Adverse effects

Among the selected studies, only the study of Moret [34] cited adverse effects. One patient reported hyperventilation complaints and had to stop traction before the end of the therapy period and another reported discomfort from the traction belt, which was easily resolved by correcting the belt position and giving extra instructions about how to fasten the belt around the chest. No other complaints were reported.

## Discussion

This systematic review aimed to investigate the effectiveness of VT in the treatment of LR. Among all the studies assessed, only three studies met the criteria to be included in our systematic review, i.e., having used VT in a well-defined population and having reported complete data.

The included studies showed large statistically significant results on pain in favor of VT only when traction was combined with a passive treatment (bed rest or medications) and compared with the same treatment alone. These results are based on very low and low quality evidence respectively. According to these results, we could infer initially that VT might be effective on pain with LR. However, when we look subsequently at the results of inversion traction combined with PT and compared with PT alone, we cannot find statistically significant results either on pain or on activity limitation. Notably, these results are based on low quality evidence.

Although pain management is a primary aim of treatment, other outcomes such as the improvement of activity limitation are relevant to a complete recovery [36]. In this study, no statistically significant results on activity limitation, albeit based on low quality evidence, were found even when VT was combined with bed rest and compared with bed rest alone.

Therefore, we conclude that the role of VT appears very limited in LR, since positive results were found only on pain and only when it was compared to medications or bed rest. Relative to the effects of medications in LR, there are conflicting conclusions on NSAIDs among who considered them as effective [8], who did not draw conclusions [6], and who did not recommend them [37, 38], while a recent systematic review suggested that corticosteroids were effective in LR [39]. Concerning the advice of bed rest or stay active in patients with sciatica, little or no difference emerged on pain and function, with moderate quality evidence [6, 40]. Therefore, better results for VT appear to emerge only when it was compared with treatments whose effectiveness is uncertain.

However, an interesting suggestion in favor of VT comes from the study of Prasad [35], where 76.9% of the patients in the traction group avoided surgery, while only 22.2% of the patient in the control group had this benefit. This might imply a relevant cost-effectiveness of VT if future research offers confirmatory evidence.

VT can be particularly appealing as a clinical tool due to some of the advantages of this kind of therapy; it is very easy to use and time sparing; and it could be applied at home more frequently and for longer duration, thereby increasing the dosage. Among the studies selected in this review, VT has been applied in very different ways. In the study of Moret [34] traction was delivered in sitting position with a belt around patient’s chest; in the study of Khani [33] patients were asked to perform an auto-traction in vertical position holding in suspension from a pull-up bar; and in the study of Prasad [35] traction was performed in inverted position. Even the ways of delivering traction force changed among the studies, with one using continuous [34] and two using intermittent [33, 35] forces. Treatment dosage was different, and control groups also received different treatments. These factors did not allow us to perform meta-analysis, so our results are based on singular analyses made per each trial.

Our results are in same direction of the systematic reviews of Cheng [41] and Zhang [39]. The first one [41] showed short-term results in favor of traction for the treatment of LBP with herniated intervertebral disks, but this review included studies on patients with and without LR. The second one [39] recommended traction in patients with radiculopathy, but it did not make any difference between patients with cervical or lumbar radiculopathy.

Other systematic reviews investigating the effectiveness of traction obtained different results, but we observe that they included studies comparing different types of traction [42–46] or the same type of traction with different force of application [47–53]. The lack of a control group, which did not receive traction, could generate a high risk of bias, especially if we assume that the effect of traction could not be only related to the delivered force [54].

Adverse effects were reported only in the study of Moret [34], but they were more related to the device than to the technique. Poor tolerance and anxiety due to inversion traction were reported by Güevenol [44]. However in this trial patients were inverted for 10 consecutive minutes, so anxiety may have been due to the treatment dosage. Static inversion may produce feelings of congestion that could be avoided delivering it in shorter periods within patient’s tolerance [55].

### Strength and limitations

Our search was extensive, using many databases and carefully consulting all published reviews and guidelines on this topic. The selection and qualitative assessment were independently done by two authors on studies reflecting clear PICOS criteria: this method minimized the heterogeneity of study population and allowed us to exclude studies with critically important missing data, and thereby reduced reference biases.

A strong publication bias is unlikely because studies in all languages, from every country and for any year of publication were included. However, we cannot exclude that we could have missed potential records, due to the search strategies we adopted. Moreover, other small studies or studies with negative studies have not published. Using only RCTs may have influenced the potential publication bias, but this approach allowed us to derive our conclusions by more rigorous studies. We believe our review has external validity because traction is often used by physical therapists for the treatment of LR, mostly in combination with treatments similar to that employed in the included trials [7, 14].

The most important limitation is related to the small number of included studies, also due to the very restricted population we considered, and the small sample sizes of included studies. It did not allow a sensitivity analysis; however, we have tried to account for the RoB found in the different studies with the GRADE method. Only two studies considered physical functioning as outcome measure, and only one study separately measured lumbar and sciatic pain.

The overall summarizing of RoB was different from current standards, having also used the “moderate risk of bias” classification, according to Gianola and colleagues [25]. The unreported standard deviations were derived from other similar studies: this may have led to an under-estimation or an over-estimation of the results.

No study was rated as “high quality,” and we did not find any published protocols, making it difficult to assess reporting bias. We cannot derive conclusions on what type of VT is better or which is the best patient’s position. Due to the heterogeneity in treatment dosage, both in terms of time of application and days of treatment, suggestions on this topic are not forthcoming.

## Conclusions

VT may be an effective treatment only for reducing pain in LR at short-term, and may be preferred to passive treatments as bed rest and medications. VT does not demonstrate significant effects on activity limitation due to LR.

We have insufficient data to conclude that VT gives additional benefits when combined to or compared with PT treatments. Further research is very likely to have an important impact on our confidence in the estimate of effect and may change these conclusions. New large, high-quality studies are needed to investigate the effectiveness of VT and identify the most effective delivering, the best treatment dosage, or the pain stage that could benefit more by this intervention.

## Supplementary Information

**Additional file 1.**

**Additional file 2.**

**Additional file 3.**

## Data Availability

The datasets used and/or analysed during the current study are available from the corresponding author on reasonable request.

## Abbreviations

CIs: Confidence Intervals
CT: Computed Tomography
GRADE: Grading of Recommendation, Assessment, Development and Evaluation
LBP: Low Back Pain
LR: Lumbar Radiculopathy
MRI: Magnetic Resonance Imaging
NRS: Numerical Rating Scale
ODI: Oswestry Disability Index
PICO: Patient, Intervention/Treatment, Control, Outcome
PT: Physical Therapy
Rayyan QRCI: Rayyan Qatar Computer Research Institute
RCT: Randomized Clinical Trial
RMDQ: Roland & Morris Disability Questionnaire
RoB: Risk of Bias
SLR: Straight Leg Raising
SMDs: Standardized Mean Differences
VAS: Visual Analogue Scale
VT: Vertical Traction
