# Supplementary material for: Vertical traction for lumbar radiculopathy: a systematic review
Source: Arch Physiother. 2021 Mar 15;11:7. doi: 10.1186/s40945-021-00102-5 (PMC7958699; doi:10.1186/s40945-021-00102-5)
Supplement: Supplementary file 2 — Additional file 2. [file 40945_2021_102_MOESM2_ESM.docx]

**APPENDIX II - SEARCH STRINGS FOR EACH DIFFERENT DATABASE**

**PubMed**

STRING:

("traction"[MeSH Terms] OR "physical therapy modalities"[MeSH Terms]) AND ("low back pain"[MeSH Terms] OR "sciatica"[MeSH Terms] OR "radiculopathy"[MeSH Terms] OR "radicular syndrome"[All Fields] OR "nerve root pain"[All Fields] OR "leg pain"[All Fields]) AND ((randomized controlled trials as topic[MeSH Terms]) OR (randomized controlled trial [All Fields] OR controlled clinical trial[All Fields] OR random*))

LIMITS:

- Species: humans
- Publication date: to 31/03/2019.

**CINHAL**

STRING:

Traction AND (low back pain OR radiculopathy OR sciatica OR leg pain OR nerve root pain OR radicular pain)

LIMITS:

- Species: humans
- Publication date: to 31/03/2019.
- Publication type: Randomized Controlled Trial

**PEDro**

Simple search: traction*

**Cochrane Library**

Advanced search 🡪 Search manager

#1 MeSH descriptor: [Traction] explode all trees

#2 MeSH descriptor: [Physical Therapy Modalities] explode all trees

#3 MeSH descriptor: [Low Back Pain] explode all trees

#4 MeSH descriptor: [Sciatica] explode all trees

#5 MeSH descriptor: [Radiculopathy] explode all trees

#6 ("radicular syndrome”):ti,ab,kw

#7 (nerve root pain):ti,ab,kw

#8 (leg pain):ti,ab,kw

#9 MeSH descriptor: [Randomized Controlled Trial] explode all trees

#10 ("randomized control trial”):ti,ab,kw

#11 ("controlled clinical trial”):ti,ab,kw

#12 random

#13 (#1 OR #2) AND (#3 OR #4 OR #5 OR #6 OR #7 OR #8) AND ((#9) OR (#10 OR #11 OR #12))

**Web of Science**

STRING (All Fields)

traction* AND (low back pain OR radicul* OR sciatica OR leg pain OR nerve root pain) AND random*

**Scopus**

STRING:

traction  AND  ("nerve root pain"  OR  radiculopathy  OR  sciatica  OR  "leg pain"  AND  "randomized controlled trial”)
