# Supplementary material for: Vertical traction for lumbar radiculopathy: a systematic review
Source: Arch Physiother. 2021 Mar 15;11:7. doi: 10.1186/s40945-021-00102-5 (PMC7958699; doi:10.1186/s40945-021-00102-5)
Supplement: Supplementary file 3 — Additional file 3. [file 40945_2021_102_MOESM3_ESM.docx]

**Appendix III**

**List of excluded full texts, with reasons according to the PICOS model (total number=91)**

**Population (n=32)**

1. Andersson G, Lucente T, Davis AM. A comparison of osteopathic spinal manipulation with standard care for patients with low back pain. N Engl J Med. 1999.
2. Aure OF, Nilsen JH, Vasselijen O. Manual Therapy and Exercise Therapy in Patients With Chronic Low Back Pain. Spine (Phila Pa 1976). 2003.
3. Beurskens AJ, de Vet HC, Koke AJ et al. Efficacy of traction for non-specific low back pain: a randomised clinical trial. Lancet. 1995 Dec 16;346(8990):1596-600.
4. Borman P et al. The efficacy of lumbar traction in the management of patients with low back pain. Rheumatol Int. 2003. PMID: 12634941.
5. Calmels P, Jacob JF, Fayolle-Minon I et al. Use of isokinetic techniques vs standard physiotherapy in patients with chronic low back pain. Preliminary results. Ann Readapt Med Phy. 2004 Feb;47(1):20-7. doi: 10.1016/j.annrmp.2003.07.001.
6. Cambron JA, Gudavalli MR, Hedeker D et al. One-Year Follow-Up of a Randomized Clinical Trial Comparing Flexion Distraction with an Exercise Program for Chronic Low-Back Pain. J Altern Complement Med. 2006 Sep;12(7):659-68. doi: 10.1089/acm.2006.12.659.PMID: 16970536
7. Carr JL, Klaber Moffet JA, Howath E et al. A randomized trial comparing a group exercise programme for back pain patients with individual physiotherapy in a severely deprived area. Disabil Rehabil. 2005 Aug 19;27(16):929-37. doi: 10.1080/09638280500030639.
8. Cecchi F, Negrini S, Pasquini G et al. Predictors of functional outcome in patients with chronic low back pain undergoing back school, individual physiotherapy or spinal manipulation. Eur J Phys Rehabil Med. 2012. PMID: 22569488 Clinical Trial.
9. Cevik R, Bilici A, Gur A et al. Effect of new traction technique of prone position on distraction of lumbar vertebrae and its relation with different application of heating therapy in low back pain. Journal of Back and Musculoskeletal Rehabilitation 20 (2007) 71–77.
10. Demirel A, Yorubulut M, Ergun N. Regression of lumbar disc herniation by physiotherapy. Does non-surgical spinal decompression therapy make difference? Double-blind randomized controlled trial. J Back Musculoskelet Rehabil. 2017 Sep 22;30(5):1015-1022. doi: 10.3233/BMR-169581.
11. Diab A, Moustafa IM. Lumbar Lordosis Rehabilitation For Pain And Lumbar Segmental Motion In Chronic Mechanical Low Back Pain: A Randomized Trial. J Manipulative Physiol Ther. 2012. PMID: 22632584 Clinical Trial.
12. Diab A, Moustafa IM. The efficacy of lumbar extension traction for sagittal alignment in mechanical low back pain: A randomized trial. J Back Musculoskelet Rehabil. 2013;26(2):213-20. doi: 10.3233/BMR-130372.
13. Fritz JM, Delitto A, Erhard RE. Comparison of Classification-Based Physical Therapy With Therapy Based on Clinical Practice Guidelines for Patients with Acute Low Back Pain. Spine (Phila Pa 1976). 2003 Jul 1;28(13):1363-71; discussion 1372. doi: 10.1097/01.BRS.0000067115.61673.FF.
14. Fritz JM, Magel JS, McFadden M et al. Early Physical Therapy vs Usual Care in Patients With Recent-Onset LowBack Pain. A Randomized Clinical Trial. JAMA. 2015 Oct 13;314(14):1459-67. doi: 10.1001/jama.2015.11648.
15. Gmünder R, Kissling R. The Efficacy of homoepathy in the treatment of chronic low back pain compared to standardized physiotherapy. Z Orthop Ihre Grenzgeb. Sep-Oct 2002;140(5):503-8.doi: 10.1055/s-2002-34004.
16. Gudavalli MR, Cambron JA, McGregor M. A randomized clinical trial and subgroup analysis to compare flexion–distraction with active exercise for chronic low back pain. Eur Spine J. 2006 Jul;15(7):1070-82. doi: 10.1007/s00586-005-0021-8. Epub 2005 Dec 8.
17. Hamre HJ, Witt CM, Glockmann A. Anthroposophic vs conventional therapy for chronic low back pain: a prospective comparative study. Eur J Med Res. 2007 Jul 26;12(7):302-10.
18. Hsieh LLC, Kuo CH, Yen MF. A randomized controlled clinical trial for low back pain treated by acupressure and physical therapy. Prev Med. 2004. PMID: 15207999 Clinical Trial.
19. Hurley DA, O'Donoghue G, Tully MA. A walking programme and a supervised exercise class versus usual physiotherapy for chronic low back pain: a single-blinded randomised controlled trial. (The Supervised Walking In comparison to Fitness Training for Back Pain (SWIFT) Trial). Prev Med. 2004 Jul;39(1):168-76. doi: 10.1016/j.ypmed.2004.01.036.
20. Hurwitz EL, Morgenstern H, Harber P. A Randomized Trial of Medical Care With and Without Physical Therapy and Chiropractic Care With and Without Physical Modalities for Patients With Low Back Pain: 6-Month Follow-Up Outcomes From the UCLA Low Back Pain Study. Spine (Phila Pa 1976). 2002 Oct 15;27(20):2193-204. doi: 10.1097/00007632-200210150-00002.
21. Koçak FA, Tunç H, Sütbeyaz ST. Comparison of the short-term effects of the conventional motor traction with non-surgical spinal decompression performed with a DRX9000TM device on pain, functionality, depression, and quality of life in patients with low back pain associated with lumbar disc herniation: A single-blind randomized-controlled trial. Turk J Phys Med Rehabil. 2017 Feb 16;64(1):17-27. doi: 10.5606/tftrd.2017.154. eCollection 2018 Mar.
22. Konrad K, Tatrai T, Hunka A, Vereckei E,. Controlled trial of balneotherapy in treatment of low back pain. Ann Rheum Dis. 1992. PMID: 1535495.
23. Letchuman R, Deusinger RH. Comparison of Sacrospinalis Myoelectric Activity and Pain Levels in Patients Undergoing Static and Intermittent Lumbar Traction. Spine (Phila Pa 1976). 1993 Aug;18(10):1361-5. doi: 10.1097/00007632-199308000-00017.
24. Licciardone JC, Minotti DE, Gatchel RJ et al. Osteopathic Manual Treatment and Ultrasound Therapy for Chronic Low Back Pain: A Randomized Controlled Trial. Ann Fam Med. 2013. PMID: 23508598 .
25. Licciardone JC, Kearns CM, Minotti DE. Outcomes of osteopathic manual treatment for chronic low back pain according to baseline pain severity: Results from the OSTEOPATHIC Trials. Man Ther. 2013 Dec;18(6):533-40. doi: 10.1016/j.math.2013.05.006. Epub 2013 Jun 10.
26. Licciardone JC, Aryal S. Clinical Response and Relapse in Patients with Chronic Low Back Pain Following Osteopathic Manual Treatment: Results from the OSTEOPATHIC Trial. Man Ther. 2014 Dec;19(6):541-8. doi: 10.1016/j.math.2014.05.012. Epub 2014 Jun 5.
27. Licciardone JC, Gatchel RJ, Aryal S. Recovery From Chronic Low Back Pain After Osteopathic Manipulative Treatment: A Randomized Controlled Trial. J Am Osteopath Assoc. 2016 Mar;116(3):144-55. doi: 10.7556/jaoa.2016.031.
28. Parkin-Smith GF, Norman IJ, Briggs E, et al. A Structured Protocol of Evidence-Based Conservative Care Compared With Usual Care for Acute Nonspecific Low Back Pain: A Randomized Clinical Trial. Arch Phys Med Rehabil. 2012 Jan;93(1):11-20. doi: 10.1016/j.apmr.2011.08.022.
29. Rhon DI, Miller RB, Fritz JM. Effectiveness and downstream healthcare utilization for patients that received early physical therapy versus usual care for low back pain: a randomized clinical trial. Spine (Phila Pa 1976). 2018. PMID: 29489568 Clinical Trial.
30. Skargren EI, Carlsson PG, Oberg BE. One-Year Follow-up Comparison of the Cost and Effectiveness of Chiropratic and Physiotherapy as Primary Management for Back Pain. Spine (Phila Pa 1976). 1998 Sep 1;23(17):1875-83; discussion 1884. doi: 10.1097/00007632-199809010-00016.
31. Schimmel JJP, de Kleuver M, Horsting PP. No effect of traction in patients with low back pain: a single centre, single blind, randomized controlled trial of Intervertebral Differential Dynamics Therapy. Eur Spine J. 2009 Dec;18(12):1843-50. doi: 10.1007/s00586-009-1044-3. Epub 2009 May 31.
32. Veihelmann A, Devens C, Trouillier H, Birkenmaier C. Epidural neuroplasty versus physiotherapy to relieve pain in patients with sciatica: a prospective randomized blinded clinical trial. J Orthop Sci. 2006 Jul;11(4):365-9. doi: 10.1007/s00776-006-1032-y.

**Intervention (n=34)**

1. Bilgilisoy Filiz M, Kiliç Z, Uçkun A et al. Mechanical Traction for Lumbar Radicular Pain: Supine or Prone? A Randomized Controlled Trial. Am J Phys Med Rehabil. 2018. PMID: 29309314 Clinical Trial.
2. Choi J, Lee S, Hwangbo G . Influences of spinal decompression therapy and general traction therapy on the pain, disability, and straight leg raising of patients with intervertebral disc herniation. J Phys Ther Sci. 2015 Feb;27(2):481-3. doi: 10.1589/jpts.27.481. Epub 2015 Feb 17.
3. Cleland JA, Fritz JM, Kulig K et al. Comparison of the Effectiveness of Three Manual Physical Therapy Techniques in a Subgroup of Patients With Low Back Pain Who Satisfy a Clinical Prediction Rule. Spine (Phila Pa 1976). 2009 Dec 1;34(25):2720-9. doi: 10.1097/BRS.0b013e3181b48809.
4. Fritz JM, Cleland JA, Childs JD. Subgrouping patients with low back pain: Evolution of a classification approach to physical therapy. J Orthop Sports Phys Ther. 2007 Jun;37(6):290-302. doi: 10.2519/jospt.2007.2498.
5. Frost H, Lamb SE, Doll HA. Randomised controlled trial of physiotherapy compared with advice for low back pain. BMJ. 2004 Sep 25;329(7468):708. doi: 10.1136/bmj.38216.868808.7C. Epub 2004 Sep 17.
6. Goldby LJ, Moore AP, Doust J. A Randomized Controlled Trial Investigating the Efficiency of Musculoskeletal Physiotherapy on Chronic Low Back Disorder. Spine (Phila Pa 1976). 2006 May 1;31(10):1083-93. doi: 10.1097/01.brs.0000216464.37504.64.
7. Hertzman-Miller RP, Morgenstern H, Hurwitz EL. Comparing the Satisfaction of Low Back Pain Patients Randomized to Receive Medical or Chiropractic Care: Results From the UCLA Low-Back Pain Study.
   Am J Public Health. 2002 Oct;92(10):1628-33. doi: 10.2105/ajph.92.10.1628.
8. Hill JC, Whitehurst DGT, Lewis M. Comparison of stratified primary care management for low back pain with current best practice (STarT Back): a randomised controlled trial. Lancet. 2011 Oct 29;378(9802):1560-71. doi: 10.1016/S0140-6736(11)60937-9. Epub 2011 Sep 28.
9. Hofstee DJ, Gijtenbeek JMM, Hoogland PH. Westeinde Sciatica Trial: randomized controlled study of bed rest and physiotherapy for acute sciatica. J Neurosurg. 2002 Jan;96(1 Suppl):45-9. doi: 10.3171/spi.2002.96.1.0045.
10. Hsieh LLC, Kuo CH, Lee LH. Treatment of low back pain by acupressure and physical therapy: randomised controlled trial. BMJ. 2006 Mar 25;332(7543):696-700. doi: 10.1136/bmj.38744.672616.AE. Epub 2006 Feb 17.
11. Hurwitz EL, Morgenstern H, Harber P. A Randomized Trial of Medical Care With and Without Physical Therapy and Chiropractic Care With and Without Physical Modalities for Patients With Low Back Pain: 6-Month Follow-Up. Spine (Phila Pa 1976). 2002 Oct 15;27(20):2193-204. doi: 10.1097/00007632-200210150-00002.
12. Hurwitz EL, Morgenstern H, Kominski GF. A Randomized Trial of Chiropractic and Medical Care for Patients With Low Back Pain. Spine (Phila Pa 1976). 2006 Mar 15;31(6):611-21; discussion 622. doi: 10.1097/01.brs.0000202559.41193.b2.
13. Jahantiqh F, Abdollahimohammad A, Firouzkouhi M et al. Effects of Reiki Versus Physiotherapy on Relieving Lower Back Pain and Improving Activities Daily Living of Patients With Intervertebral Disc Hernia. J Evid Based Integr Med. Jan-Dec 2018;23:2515690X18762745. doi: 10.1177/2515690X18762745.
14. Kotb HA, Effat DA, Awad MR, et al. CT-guided transforaminal epidural steroid injection and vertebral axial decompression in management of acute lumbar disc herniation. The Egyptian Rheumatologist (2017), http://dx.doi.org/10.1016/j.ejr.2017.04.012.
15. Licciardone JC, Aryal S. Clinical Response and Relapse in Patients with Chronic Low Back Pain Following Osteopathic Manual Treatment: Results from the OSTEOPATHIC Trial. Man Ther. 2014 Dec;19(6):541-8. doi: 10.1016/j.math.2014.05.012. Epub 2014 Jun 5.
16. Louw A, Farrell K, Landers M et al. The effect of manual therapy and neuroplasticity education on chronic low back pain: a randomized clinical trial. J Man Manip Ther. 2017 Dec;25(5):227-234. doi: 10.1080/10669817.2016.1231860. Epub 2016 Sep 22.
17. Moustafa IM, Diab AA. Extension traction treatment for patients with discogenic lumbosacral radiculopathy: a randomized controlled trial. Clin Rehabil. 2013 Jan;27(1):51-62. doi: 10.1177/0269215512446093. Epub 2012 Jun 8.
18. Murtezani A, Hundozi H, N Orovcanec N. A comparison of high intensity aerobic exercise and passive modalities for the treatment of workers with chronic low back pain: a randomized controlled trial. Eur J Phys Rehabil Med. 2011 Sep;47(3):359-66. Epub 2011 May 23.
19. Nikoobakht M, Yekanineajd MS, Pakpour AH. Plasma disc decompression compared to physiotherapy for symptomatic contained lumbar disc herniation: A prospective randomized controlled trial. Neurol Neurochir Pol. 2016;50(1):24-30. doi: 10.1016/j.pjnns.2015.11.001. Epub 2015 Nov 28.
20. Ozturk B, Gunduz OH, Ozoran K. Effect of continuous lumbar traction on the size of herniated disc material in lumbar disc herniation. Rheumatol Int. 2006 May;26(7):622-6. doi: 10.1007/s00296-005-0035-x. Epub 2005 Oct 25.
21. Paatelma M, Kilpikoski S, Simonen R. Orthopaedic manual therapy, McKenzie method or advice only for low back pain in working adults: a randomized controlled trial with one year follow-up. J Rehabil Med. 2008 Nov;40(10):858-63. doi: 10.2340/16501977-0262.
22. Peul WC, van Houwelingen HC, van den Hout WB. Surgery versus Prolonged Conservative Treatment for Sciatica. N Engl J Med. 2007 May 31;356(22):2245-56.
23. Peul WC, van den Hout WB, Brand R. Prolonged conservative care versus early surgery in patients with sciatica caused by lumbar disc herniation: two year results of a randomised controlled trial. BMJ. 2008 Jun 14;336(7657):1355-8. doi: 10.1136/bmj.a143. Epub 2008 May 23.
24. Rasmussen-Barr E, Nilsson-Wikmar L, Arvidsson I. Stabilizing training compared with manual treatment in sub-acute and chronic low-back pain. Man Ther. 2003 Nov;8(4):233-41. doi: 10.1016/s1356-689x(03)00053-5.
25. Seferlis T, Németh G, Carlsson AM. Conservative treatment in patients sick-listed for acute low-back pain: a prospective randomised study with 12 months’ follow-up. Eur Spine J. 1998;7(6):461-70. doi: 10.1007/s005860050109.
26. Sherry E, Kitchener P, Smart R. A prospective randomized controlled study of VAX-D and TENS for the treatment of chronic low back pain. Neurol Res. 2001 Oct;23(7):780-4. doi: 10.1179/016164101101199180.
27. Simmermann SM, Sizer PS, Dedrick GS et al. Immediate Changes in Spinal Height and Pain After Aquatic Vertical Traction in Patients With Persistent Low Back Symptoms: A Crossover Clinical Trial. PM R. 2011 May;3(5):447-57. doi: 10.1016/j.pmrj.2011.01.010.
28. Skargren EI, Oberg BE, Carlsson PG et al. Cost and Effectivenes Analysis of Chiropratic and Physiotherapy treatment for Low Back and Neck Pain. Spine (Phila Pa 1976). 1997 Sep 15;22(18):2167-77. doi: 10.1097/00007632-199709150-00015.
29. Szulc P, Lewandowski J, Boch-Kmieciak J et al. The objective evaluation of effectiveness of manual treatment of spinal function disturbances. Med Sci Monit. 2012 May;18(5):CR316-22. doi: 10.12659/msm.882738.
30. Thackeray A, Fritz JM, Childs JD et al. The effectiveness of mechanical traction among subgroups of patients with low back pain and leg pain: a randomized trial. J Orthop Sports Phys Ther. 2016 Mar;46(3):144-54. doi: 10.2519/jospt.2016.6238. Epub 2016 Jan 26.
31. Unlu Z, Tasci S, Tarhan S et al. Comparison of 3 physical therapy modalities for acute pain in lumbar disc herniation measured by clinical evaluation and magnetic resonance imaging. J Manipulative Physiol Ther. 2008 Mar;31(3):191-8. doi: 10.1016/j.jmpt.2008.02.001.
32. Van den Hout VB, Peul WC, Koes BW. Prolonged conservative care versus early surgery in patients with sciatica from lumbar disc herniation: cost utility analysis alongside a randomised controlled trial. BMJ. 2008 Jun 14;336(7657):1351-4. doi: 10.1136/bmj.39583.709074.BE. Epub 2008 May 23.
33. Verwoerd AJH, Luijsterburg PAJ, Koes BW et al. Does Kinesiophobia Modify the Effect of Physical Therapy on Outcome in Patients With Sciatica in Primary Care? Subgroup Analysis From a Randomized Controlled Trial. Phys Ther. 2015 Sep;95(9):1217-23.doi: 10.2522/ptj.20140458. Epub 2015 Apr 30.
34. Wilkey A, Gregory M, Byfield D et al. A Comparison Between Chiropractic Management and Pain Clinic Management for Chronic Low-Back Pain in a National Health Service Outpatient Clinic. J Altern Complement Med. 2008 Jun;14(5):465-73. doi: 10.1089/acm.2007.0796.

**Comparison (n=12; 7=different force; 5=different traction type)**

**DIFFERENT FORCE**

1. Isner-Horobeti ME, Dufour SP, Schaeffer M et al. High-Force Versus Low-Force Lumbar Traction in Acute Lumbar Sciatica Due to Disc Herniation: A Preliminary Randomized Trial. J Manipulative Physiol Ther. Nov-Dec 2016;39(9):645-654. doi: 10.1016/j.jmpt.2016.09.006. Epub 2016 Nov 9.
2. Mathews JA, Hickling J. Lumbar traction: a double blind controlled study for sciatica. Rheumatol Rehabil. 1975 Nov;14(4):222-5. doi: 10.1093/rheumatology/14.4.222.
3. Murat S, Uzunka K, Erden N. The effect of traction with two different load on clinic and functional status of patients with subacute lumbar disc herniation. Medeniyet Medical Journal 2018;33(2):82-88 doi:10.5222/MMJ.2018.34711.
4. Pal B, Mangion P, Hossain MA, Diffey BL. A controlled trial of continuous lumbar traction in the treatment of back pain and sciatica. Br J Rheumatol. 1986 May;25(2):181-3. doi: 10.1093/rheumatology/25.2.181.
5. Reust P, Chantraine A, Vischer TL. Treatment of lumbar sciatica with or without neurological deficit using mechanical traction. A double-blind study. Schweiz Med Wochenschr. 1988 Feb 27;118(8):271-4.
6. Van Der Heijden GJMG, Beurskens A, Dirx MJM et al. Efficacy of lumbar traction: a randomised clinical trial. Physiotherapy. 1995 Jan 31; 81, 29-35. https://doi.org/10.1016/s0031-9406(05)67032-0.
7. Weber H. Traction therapy in sciatica due to disc prolapse (does traction treatment have any positive effect on patients suffering from sciatica caused by disc prolapse?. J Oslo City Hosp. 1973 Oct;23(10):167-76.

**DIFFERENT TYPE**

1. Choi J, Lee S, Hwangbo G. Influences of spinal decompression therapy and general traction therapy on the pain, disability, and straight leg raising of patients with intervertebral disc herniation. J Phys Ther Sci. 2015 Feb;27(2):481-3.doi: 10.1589/jpts.27.481. Epub 2015 Feb 17.
2. Guvenol K, TuÈzuÈn C, Peker O et al. A comparison of inverted spinal traction and conventional traction in the treatment of lumbar disc Herniations. Physiotherapy Theory an d Practice (2000) 16, 151 –160.
3. Mathewes JA, [Hickling](https://pubmed-ncbi-nlm-nih-gov.ezproxy.unibo.it/?term=Hickling+J&cauthor_id=1105752) J. Lumbar traction: a double-blind controlled study for sciatica. Rheumatol Rehabil. 1975 Nov;14(4):222-5. doi: 10.1093/rheumatology/14.4.222.
4. Tesio L, Merlo A. Autotraction versus passive traction: an open controlled study in lumbar disc herniation. Arch Phys Med Rehabil. 1993 Aug;74(8):871-6. doi: 10.1016/0003-9993(93)90015-3.
5. Weber H, Ljunggren AE, Walker L. Traction therapy in patients with herniated lumbar intervertebral discs. J Oslo City Hosp. Jul-Aug 1984;34(7-8):61-70.

**Outcome (n=1)**

1. Ljunggren AE, Walker L, Weber H, Amundsen T. Manual traction versus isometric exercises in patients with herniated intervertebral lumbar discs. Physiotherapy Theory and Practice 1992;8(4):207-213.

**Study design (n=11)**

1. Cambron JA, Gudavalli MR, Hedeker D et al. One-Year Follow-Up of a Randomized Clinical Trial Comparing Flexion Distraction with an Exercise Program for Chronic Low-Back Pain. J Altern Complement Med. 2006 Sep;12(7):659-68. doi: 10.1089/acm.2006.12.659.
2. Clarke JA, van Tulder MW, Blomberg SEI et al. Traction for low-back pain with or without sciatica. Cochrane Database Syst Rev. 2005 Oct 19;(4):CD003010. doi: 10.1002/14651858.CD003010.pub3.
3. Fritz JM, Thackeray A, Childs JD et al. A randomized clinical trial of the effectiveness of mechanical traction for sub-groups of patients with low back pain: study methods and rationale. BMC Musculoskelet Disord. 2010 Apr 30;11:81. doi: 10.1186/1471-2474-11-81.
4. Harte AA, Baxter GD, Gracey JH. The effectiveness of motorised lumbar traction in the management of LBP with lumbo sacral nerve root involvement: a feasibility study. BMC Musculoskelet Disord. 2007 Nov 29;8:118.doi: 10.1186/1471-2474-8-118.
5. Knuttson E, Skoglund CR, Natchev E. Changes in voluntary muscle strength, somatosensory transmission and skin temperature concomitant with pain relief during autotraction in patients with lumbar and sacral root lesions.
   Pain. 1988 May;33(2):173-9.doi: 10.1016/0304-3959(88)90088-7.
6. Lind GA. Auto‐traction, Treatment of Low Back Pain and Sciatica. An Electromyographic, Radiographic and Clinical Study. Thesis. University Med Diss, Linkopings 1974.
7. Luk’ianov VN,  [Prokopets](https://pubmed-ncbi-nlm-nih-gov.ezproxy.unibo.it/?term=Prokopets+NA&cauthor_id=4722263) NA. Treatment of diskogenic lumbo-sacral and thoracic radiculitis by the method of underwater spinal traction. Vrach Delo. 1973 Jan;1:116-8.
8. Ma SY, Kim HD. The efficacy of spinal decompression via DRX3000 combined with a spinal mobilization and a lumbar stabilization exercise program for patients with discogenic low back pain. Journal of Physical Therapy Science. 2010. Volume 22 Issue 4 Pages 345-354
9. Mathews W, Morkel M, Mathews J. Manipulation and traction for lumbago and sciatica: physiotherapeutic techniques used in two controlled trials. Physiotherapy Practice. 1998. 4, 201-206.
10. Olah M, Molnár L, Dobai J et al. The effects of weightbath traction hydrotherapy as a component of complex physical therapy in disorders of the cervical and lumbar spine: a controlled pilot study with follow-up. Rheumatol Int. 2008 Jun;28(8):749-56.doi: 10.1007/s00296-008-0522-y. Epub 2008 Jan 12.
11. Yang HS, Yoo WG. The effects of stretching with lumbar traction on VAS and Oswestry scales of patients with lumbar 4-5 herniated intervertebral disc. J Phys Ther Sci. 2014 Jul;26(7):1049-50.doi: 10.1589/jpts.26.1049. Epub 2014 Jul 30.

**Duplication of results (n=1)**

1. Oláh M, Molnár L, Dobai J et al. The effects of infrared laser therapy and weightbath traction hydrotherapy as components of complex physical treatment in disorders of the lumbar spine: a controlled pilot study with follow-up. Rheumatol Int. 2008 Jun;28(8):749-56.doi: 10.1007/s00296-008-0522-y. Epub 2008 Jan 12.
